# Fusing EEG Features Extracted by Microstate Analysis and Empirical Mode Decomposition for Diagnosis of Schizophrenia

**DOI:** 10.3390/s26020727

**Published:** 2026-01-21

**Authors:** Shirui Song, Lingyan Du, Jie Yin, Shihai Ling

**Affiliations:** 1 School of Automation and Information Engineering, Sichuan University of Science and Engineering, Zigong 643000, China; 2 Intelligent Perception and Control Key Laboratory of Sichuan Province, Yibin 644000, China

**Keywords:** electroencephalography, schizophrenia classification, machine learning, microstate, empirical mode decomposition

## Abstract

Accurate early diagnosis and precise assessment of disease severity are imperative for the treatment and rehabilitation of schizophrenia patients. To achieve this, we propose a computer-aided diagnostic method for schizophrenia that utilizes fusion features derived from microstate analysis and empirical mode decomposition (EMD) based on Electroencephalography (EEG) signals. At the same time, the obtained fusion features from microstate analysis and EMD are input into the Least Absolute Shrinkage and Selection Operator (LASSO) feature selection algorithm to reduce the dimensionality of feature vectors. Finally, the reduced feature vector is fed to a Logistic Regression classifier to classify SCH and healthy EEG signals. In addition, the ability of the integrated features to distinguish the severity of schizophrenia symptoms was evaluated, and the Shapley Additive Explanations (SHAP) algorithm was used to analyze the importance of the classification features that differentiate schizophrenia symptoms. Experimental results from both public and private datasets demonstrate the efficacy of EMD features in identifying healthy controls, while microstate features excel in classifying the severity of symptoms among schizophrenia patients. The classification evaluation metrics of the fused features significantly outperform those obtained using EMD or microstate analysis features independently. The fusion feature method proposed in this study achieved accuracies of 100% and 90.7% for the classification of schizophrenia in public datasets and private datasets, respectively, and an accuracy of 93.6% for the classification of schizophrenia symptoms in private datasets.

## 1. Introduction

Schizophrenia (SCZ) is a profound psychiatric disorder characterized by cognitive impairments, affective disturbances, and disorganized thought processes. The clinical diagnosis of SCZ is based on the behavioral phenotypic characteristics outlined in the Diagnostic and Statistical Manual of Mental Disorders (DSM-V) [1] or the International Classification of Diseases 11th Revision (ICD-11) [2]. The severity of the patient’s symptoms was evaluated utilizing the Brief Psychiatric Rating Scale (BPRS) [3]. According to clinical diagnoses [4], individuals with a BPRS score exceeding 53 typically exhibit severe symptoms and may necessitate more intensive and aggressive treatment interventions, whereas those with scores below 53 generally present with relatively mild symptoms. In clinical practice, the diagnosis of SCZ by physicians is predominantly based on behavioral assessments of clinical symptoms and direct patient interviews [5]. Therefore, there is an urgent need to develop a diagnostic method that possesses objective clinical significance. Electroencephalography (EEG) serves as a robust technique for evaluating cerebral activity by quantifying neural oscillations that signify large scale synchronization within the human brain in response to external stimuli [6,7]. This technique enhances the analysis of neural synchrony relevant to the pathophysiology of schizophrenia and has emerged as a crucial tool for identifying and diagnosing pathological or psychological states within the brain [8]. Furthermore, the temporal dynamics of brain function can be elucidated through the dynamic characteristics of EEG data [9], which aids in elucidating the underlying mechanisms of schizophrenia.

A large number of studies have extracted features from EEG data to detect patients with SCZ. Racz et al. [10] achieved an accuracy of 89% by using dynamic functional networks and random forests; Khare et al. [11] combined smoothed pseudo-Wigner-Ville distribution (SPWVD) with CNN and achieved an accuracy of 93.96%; Kumar et al. [12] used symmetrically weighted local binary patterns (SLBP) and AdaBoost to achieve an accuracy of up to 99.36%. As research on feature extraction continues to advance, the simplification of data feature extraction methods, enhancement of interpretability and clinical recognition of features, and reduction in the dimensionality of classification-relevant features have become focal points for many researchers. De Miras et al. [13] extracted nonlinear features from resting-state EEG data and used a support vector machine based on radial basis function (SVM-RBF) for classification, achieving an average accuracy rate of 92.91%. Subsequently, Krishnan et al. [14] employed multivariate empirical mode decomposition (MEMD) to analyze EEG signals and used the SVM-RBF classifier for evaluation, with an accuracy rate and F1 score reaching 93%. Lillo et al. [15] analyzed EEG signals after microstate analysis using a random walk strategy, combined with a CNN for feature extraction, and achieved a classification accuracy rate of 93% using the selected dataset. Meanwhile, Yan et al. [16] extracted visual behavioral features and physiological features from driver facial images and electrocardiogram (ECG) signals, used the YOLOv4 convolutional neural network for facial state recognition, and fused the ECG signal for bimodal decision-making. Ultimately, the system achieved a maximum accuracy rate of 99% in fatigue driving detection.

Recent research [17,18,19,20] has demonstrated that nuanced alterations in brain function can be identified through EEG microstate analysis. EEG microstate analysis, characterized by its high temporal and spatial resolution, enables the decomposition of EEG signals into a series of transient and stable spatiotemporal patterns [21], which are believed to represent the brain’s instantaneous functional state at specific moments in time. In contrast to traditional EEG analysis techniques, microstate analysis provides enhanced spatiotemporal dynamic insights by effectively capturing the brain’s instantaneous activity. This information holds substantial importance in the investigation of neuropsychiatric disorders, particularly schizophrenia. Numerous studies [22,23,24,25,26] have shown that individuals with schizophrenia exhibit marked differences in transition probabilities, mean durations, time coverage, and occurrence of microstates compared to healthy controls. Nevertheless, EEG signals demonstrate both nonlinear and non-stationary properties; thus, exclusive reliance on linear analytical methods may prove insufficient in accurately capturing the intricate dynamics inherent to EEG signals. In recent years, nonlinear techniques such as Empirical Mode Decomposition (EMD) have been increasingly applied to EEG data analysis. EMD is an adaptive signal decomposition method that effectively separates complex nonlinear EEG signals into a series of IMFs, thereby facilitating the extraction of essential signal features across various temporal scales. EMD demonstrates significant efficacy in the analysis of nonstationary and nonlinear signals [27], which renders it particularly well-suited for the examination of EEG data in individuals diagnosed with SCZ. In the realm of SCZ detection research, microstate analysis offers valuable spatiotemporal insights into brain function, while EMD enhances the extraction of critical nonlinear information through signal decomposition. The synergistic fusion of these two methodologies demonstrates considerable potential.

In EEG microstate analysis, features derived from deep learning frequently lack interpretability, this study utilizes four distinct types of microstate parameter features that offer enhanced clinical explanatory significance within the medical domain. In EMD research, while features derived from entropy measures demonstrate superior classification performance, they are associated with high computational complexity during the feature extraction process. Consequently, this study presents a fusion strategy that concatenates microstate features with EMD features to classify SCZ patients from healthy controls and assess the severity of the condition in individuals affected by SCZ and employing the SHAP algorithm to elucidate the distinct contributions of EMD features and microstate features in the classification of SCZ symptoms.

## 2. Materials and Methods

### 2.1. Dataset

This study utilized two datasets: Dataset-1, which is publicly available [28], and Dataset-2, a proprietary dataset.

Dataset-1: The dataset comprised 14 patients diagnosed with schizophrenia and 14 healthy control subjects. The two sets of data were strictly matched in terms of demographic variables such as age and gender.

Dataset-2: A total of 92 patients diagnosed with chronic schizophrenia were recruited from the Department of Psychiatry at Zigong Fifth People’s Hospital, comprising 41 individuals with a BPRS score exceeding 53 and 51 individuals with a BPRS score below this threshold. All patients were diagnosed by qualified physicians utilizing the Structured Clinical Interview for DSM-IV Disorders (SCID) [29] and subsequently admitted to the hospital. The healthy control group, consisting of individuals with no prior history of mental illness and abstaining from the use of sedatives, hypnotics, or central nervous system drugs within one month prior to enrollment, was recruited from both hospitals and the community, totaling 32 participants. Recruited participants (including both patients and healthy controls) were aged 18 years or older, right-handed, native Chinese speakers, and had normal or corrected visual acuity. The inclusion criteria for all subjects were as follows: (i) a minimum of four years of education, (ii) no history of head trauma or serious physical illness, (iii) no history of substance abuse (e.g., alcoholism), (iv) absence of significant auditory-visual impairment, and (v) negative results on female urine pregnancy test to exclude pregnancy or contraindications. Electrode positioning adhered to the international 10–20 system, with auxiliary electrodes placed to aid in the localization and detection of artifacts. The sampling frequency was set at 250 Hz, and a total of 15 min of resting-state EEG data were recorded across 19 channels from the subjects. This study received ethical approval from the Ethics Committee of Zigong Fifth People’s Hospital, and all participants provided either oral consent or signed informed consent forms, in strict adherence to the principles set forth in the Declaration of Helsinki. Socio-demographic data, including age, and gender for all participants, were gathered (Table 1). BPRS was employed to evaluate the severity of the disease and its associated symptoms, with assessments conducted by two professional clinicians.

### 2.2. Electroencephalography Feature Extraction

#### 2.2.1. EEG Signal Pre-Processing

To acquire artifact-free EEG data suitable for empirical mode decomposition and microstate analysis, we employed EEGLAB (EEGLAB v2021.0) [30] within the MATLAB (MATLAB R2021a) environment to preprocess both datasets. The steps are as follows: (1) Data import: Import the collected offline (.edf file) data into EEGLAB; (2) Electrode positioning: Position the electrodes according to the international 10–20 system for the data; (3) Eliminate unutilized electrodes: Eliminate the electrode channels used for positioning during the recording, and retain 19 channels; (4) Filtering: Perform band-pass filtering within the range of 0.1 to 40 Hz, and set the notch filter to 50 Hz to eliminate power frequency interference; (5) Downsampling: Set the data sampling rate to 250 Hz; (6) Interpolation for bad channels: After manual identification of the damaged channels, use the spline interpolation algorithm to correct them; (7) Elimination of bad segments: Visual inspection to remove time segments with excessive drift or unclear recordings; (8) Re-reference: Apply bilateral mastoid reference to the EEG data of all subjects; (9) Artifact correction: Perform ICA correction for eye movement, blinking artifacts and electrocardiogram artifacts, and remove them; (10) Data quality check: Check the processed electroencephalogram data, re-identify artifact components or segments with excessive drift, remove them, and check whether the formats of all data are consistent, and save them as .set format files for subsequent micro-state analysis.

#### 2.2.2. Microstate Analysis

Microstate analysis is a methodological approach employed to analyze EEG signals, fundamentally based on the construction of transient spatial patterns derived from these signals, referred to as microstates.

Conduct microstate analysis. After data preprocessing is completed, calculate the Global Field Power (GFP) for the EEG data corresponding to each subject. That is, for each sampling point, a GFP value will be obtained. Eventually, each subject will obtain a single-channel signal composed of GFP values. Then, calculate the peak points of all GFP values with the strongest local field strength. Use the quasi-steady-state topographic maps corresponding to these sampling points as input samples for the clustering algorithm. The GFP calculation formula is as shown in Equation (1). For each participant, a total of 1000 GFP peaks were randomly selected. Subsequently, the template derived from these GFP peaks was fed into a K-Means clustering algorithm, facilitating the identification of microstate categories at each time point across all subjects’ data, thus achieving comprehensive categorization of all EEG microstates. For each subject, four index parameters were computed for each of the four microstate classes: mean duration, time coverage, occurrences, and transition probability.

(1) $$GFP\left(n\right)=\sqrt{(\sum_{i}^{N}{\left(x_{i}\left(n\right)-\bar{x}\left(n\right)\right)}^{2})/N}$$

where $x_{i}\left(n\right)$ and ${\bar{x}}_{i}\left(n\right)$ represent the instantaneous and mean potentials across *N* electrodes at time *n*.

#### 2.2.3. Empirical Mode Decomposition

EMD is an adaptive signal processing technique driven by data, primarily employed for the analysis of nonlinear and non-stationary signals [31]. The core idea centers on decomposing a complex signal into multiple intrinsic mode functions (IMFs), with each IMF capturing the oscillatory characteristics of the signal across diverse temporal scales.

In this study, the number of IMFs obtained from the EMD of EEG data ranged from nine to twelve across all subjects. The count of IMFs for each participant was standardized to nine in order to mitigate discrepancies in feature dimensions resulting from interindividual variations in IMF counts. Subsequently, fourteen conventional statistical features were computed for each IMF. The features examined encompass the mean, extreme values, quartiles and their interquartile range, median, variance, standard deviation, kurtosis, skewness, root mean square error, and natural activity.

### 2.3. Feature Level Fusion

During the preprocessing stage, both datasets were re-referenced using bilateral mastoid electrodes. Consequently, feature extraction was performed only on the 17 channels, excluding the electrode channels used for bilateral mastoid referencing. In the feature extraction process of microstate analysis, the EEG signals of each participant were clustered into four classes (A, B, C, D), with each class containing three parameters: mean duration, occurrence, and coverage, resulting in a total of 12 extracted features. Additionally, 12 transition probability features between different microstate classes were calculated, resulting in a total of 24 microstate features. As described in Section 2.2.2, the EEG signal features extracted using microstate analysis include two-dimensional features (microstate class × feature parameters) and transition probabilities. During the feature extraction process using EMD, the 17 channels of each subject were decomposed, with each channel producing 9 IMFs components. For each IMF component, 14 traditional statistical features were calculated, resulting in a total of 2142 features. As described in Section 2.2.3, the EEG signal features extracted through the EMD method are represented as three-dimensional features (N × M × C), where N denotes the number of channels, M denotes the number of IMFs, and C denotes the number of different statistical features.

**Figure 1:**
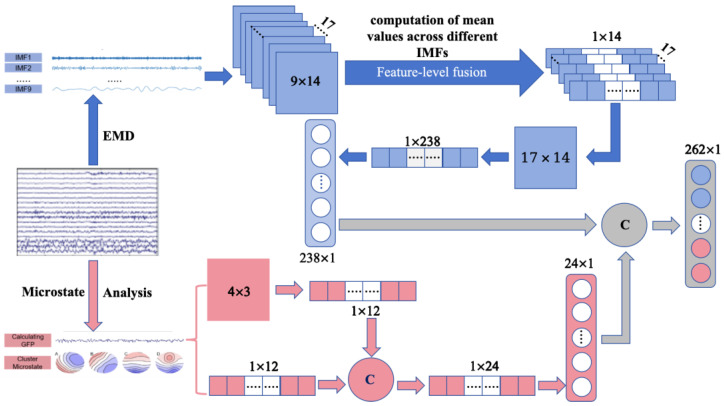
The processing steps of the methodology for Feature-Level Fusion. Here, C represents the fused features. The blue section represents the extraction of features using EMD technology, the pink section represents the extraction of features using microstate analysis, the gray section indicates feature fusion, and the arrows represent the process steps.

This study employed a method to effectively reduce the dimensionality of EMD features while aiming to minimize computational costs, followed by feature-level fusion with microstate features. The fusion mode (Figure 1) is defined as R = W + E, where R represents the fused feature, W denotes the microstate feature, and E signifies the EMD feature.

The formula for feature-level fusion is as follows (2). The features from a single subject’s EEG channel, represented as M × C, were extracted, followed by the computation of mean values across different IMFs without altering the positional integrity of the feature set, yielding a total feature count of N × C and facilitating dimensionality reduction. Using the feature fusion method, a total of 262 features were obtained.

(2) $$R=W+E_{avg}^{\left(N\times C\right)}\;where\;E_{avg}^{\left(N\times C\right)}=\left\{\frac{1}{M}\sum_{i=1}^{N}e_{i,j,k}\left|j\in \left[1,N\right],k\in \left[1,C\right]\right.\right\}$$

*R*, Fused features; *W*, Microstate features; $E_{avg}^{\left(N\times C\right)}$ Averaged EMD features, dimensions *N* × *C*; *N*, Number of channels; *M*, Number of IMF components; *C*, Number of statistical features; $e_{i,j,k}$ value of the *i*-th channel, *j*-th IMF, and *k*-th statistical feature.

### 2.4. Machine Learning and Classification

A comparative analysis of various features was conducted for two classification detection tasks (task 1: classification of schizophrenia, task 2: classification of schizophrenia symptom severity). Task 1 utilizes the entirety of Dataset-1 and a subset of Dataset-2. To ensure balance and comprehensive representation of most samples in Dataset-2, 32 patients with schizophrenia (16 cases above and below the BPRS score of 53) and 32 healthy controls were randomly selected from this dataset. For Task 2, the complete Dataset-2 was utilized, comprising 41 patients with schizophrenia exhibiting BPRS scores above 53, 51 patients with schizophrenia exhibiting BPRS scores below 53, and 32 healthy controls.

#### 2.4.1. Classifiers and Parameter Tuning

In this study, we employed the Logistic Regression classifier to evaluate the efficacy of various feature types, including individual features, and features at different levels of fusion, in the classification of schizophrenia. Multinomial Logistic Regression [32] is an extension of logistic regression tailored for multi-class classification tasks. It operates by fitting a Softmax function to map the output of linear regression to the probability values associated with each category.

In machine learning, optimizing the parameters of a classifier is a critical step in enhancing model performance. In this study, the parameters of various classifiers were optimized using the Hyperopt algorithm [33], which is based on Bayesian optimization and random search. In comparison to traditional parameter optimization methods, Hyperopt employs Bayesian optimization—specifically the Tree-structured Parzen Estimator (TPE) algorithm—to identify the optimal parameter combination in fewer iterations, thereby achieving greater efficiency.

#### 2.4.2. Feature Selection and Evaluation Metrics

**Figure 2:**
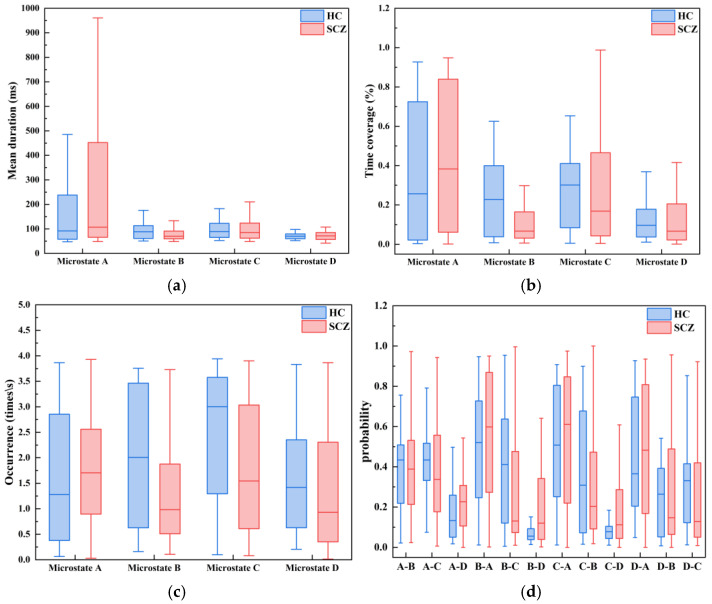
Visualization of Microstate Features. Panel (**a**) represents the mean duration, panel (**b**) represents the time coverage, panel (**c**) represents the occurrence rate, and panel (**d**) represents the transition probability.

As detailed in Section 2.3, the microstate analysis yields 24 features, while EMD results in 2142 features. Additionally, feature-level fusion scheme produces 262 features. Following the acquisition of microstate features, feature visualization was performed. As shown in Figure 2, panel (a) indicates that the mean duration of microstate class A in schizophrenia patients is significantly greater than that in healthy controls; panel (b) shows that the time coverage of microstate class A in schizophrenia patients is significantly greater than that in healthy controls; panel (c) reveals that the occurrence rate of microstate class B in schizophrenia patients is significantly lower than that in healthy controls; and panel (d) demonstrates that the transition rate from microstate class B to microstate class D in schizophrenia patients is significantly greater than that in healthy controls.

Not all features hold equal significance in classification tasks, nor do they uniformly contribute positively to the evaluation metrics; thus, feature selection is a critical step in the classification process. To reduce the complexity of the model, improve the computational efficiency, and enhance the prediction accuracy of the model, this study applied the Least Absolute Shrinkage and Selection Operator (LASSO) [34] (Equation (3)) method for feature selection. The core of the LASSO algorithm lies in introducing the L1 penalty term in the loss function, which enables the constraint and compression of the variable weights in the model.

(3) $${min}_{\omega }\sum_{i=1}^{m}\left({\left(y_{i}-\omega^{T}x_{i}\right)}^{2}+\lambda {∥\omega ∥}_{1}\right)$$

The parameters in the formula are defined as follows: ω represents the parameter or weight vector of the model; m is the number of data points or samples in the dataset; $y_{i}$ is the actual value (target or response variable) for the $i_{th}$ sample; $x_{i}$ is the feature vector for the $i_{th}$ sample; $\omega^{T}x_{i}$ is the predicted value, λ controls the strength of L1 regularization, and ${∥\omega ∥}_{1}$ is the L1 norm that promotes sparsity by shrinking some coefficients to zero.

In this study, a subject-based 10-fold cross-validation (10-CV) was utilized to enhance the generalization capability of the classification model and reduce the risk of data leakage. The dataset is partitioned on a per-subject basis to ensure that all data pertaining to each individual is exclusively assigned either to the training set or the test set, without any overlap between them. The dataset was divided into 10 subsets, with one subset randomly selected as the test set in each iteration, while the remaining nine subsets were utilized to train the model. Meanwhile, LASSO feature selection was performed in the training set of each cross-validation fold, to ensure that the “data leakage” phenomenon does not occur. This process was repeated 10 times, and the final classification performance metrics were averaged from the results of these ten experiments to yield an overall assessment of the model’s performance. The evaluation metrics employed in this study included the Area Under the Receiver Operating Characteristics Curve (AUC), Accuracy (ACC), Specificity (SPE), and Sensitivity (SEN).

### 2.5. Shapley Additive Explanations

The SHAP algorithm [35], grounded in Shapley values from cooperative game theory, offers a method for elucidating the outputs of machine learning models. SHAP assigns each feature a contribution value that quantifies its impact on the model’s predictions. The core principle involves computing the marginal contribution of each feature by evaluating all possible combinations of features, thereby ensuring consistency and fairness; this means that the contribution of a feature remains stable regardless of the order in which features are incorporated. SHAP is particularly advantageous as it provides a unified framework for both global feature importance and local feature contributions to individual predictions, rendering it a widely utilized tool in model interpretability.

## 3. Result and Discussion

This section presents the research results and corresponding discussions regarding the proposed method in this paper for optimizing feature extraction and its fusion features in two classification tasks for schizophrenia. Simultaneously, the classification performance of the proposed method is compared with that of contemporary and relatively novel classification techniques for schizophrenia EEG signals.

### 3.1. Classification Between Schizophrenia Patients and Healthy Controls

In the classification of schizophrenia, we initially demonstrate the efficacy of the feature-level fusion strategy proposed in this study. Table 2 presents the classification evaluation metrics for the three features in Dataset-1 as assessed by the logistic regression classifier. In Dataset-1, both EMD features and microstate features demonstrated excellent classification performance with an AUC of 1, indicating their exceptionally high sensitivity and accuracy in differentiating schizophrenia patients from healthy controls. The specificity of the micro-state features was 0.900 and the sensitivity was 1, while the specificity of the EMD features was 0.950 and the sensitivity was 1. The feature-level fusion method significantly improved the classification performance. Feature-Level Fusion achieved perfect scores of 1 in all indicators. This underscores the effectiveness of integrating diverse features and provides robust support for the classification of schizophrenia. However, given the limited sample size in Dataset-1, which may not adequately represent the entire spectrum of symptoms, we conduct repeated validation on Dataset-2 to assess the robustness of our method.

In the evaluation metrics of Dataset 2 (Table 3), the AUC value of EMD features is 0.916, indicating that its classification ability remains robust, but it has decreased compared to Dataset 1; the specificity and sensitivity are 0.917 and 0.875, respectively. The performance of microstate features is poor, with an AUC value of 0.758, and the specificity and sensitivity are 0.592 and 0.817 respectively. This indicates inherent limitations in identifying schizophrenia. However, feature-level fusion performed strongly on Dataset 2, achieving an AUC value of 0.972, while maintaining high specificity and sensitivity. These findings further highlight the importance of feature fusion in the classification of schizophrenia.

By synthesizing the evaluation metrics from both datasets, we observe that while the metrics for Dataset-1 are superior to those of Dataset-2, the feature-level fusion strategy exhibits significant advantages in both datasets. This demonstrates that the fusion of diverse features can significantly enhance the accuracy and reliability of schizophrenia classification, with the feature fusion method representing one of the effective strategies to improve classification performance. Simultaneously, the significance of methodology validation across diverse datasets is underscored.

**Table 4:** Comparison of the previous approaches for SZ detection with our work.

Reference NO.	Feature Extraction	Number of Subjects	Classification Algorithm	ACC(%)
Krishnan et al. [14] (2020)	Multivariate Empirical mode decomposition Entropy	Healthy:14, SZ:14	SVM-RBF	93
Kim et al. [36] (2021)	Conventional + Microstates Feature	Healthy:14, SZ:14	SVM	76.85
Aslan et al. [37] (2022)	CWT-based scalogram + Image augmentation	Healthy:14, SZ:14	CNN	99.50
Lillo et al. [15] (2022)	Microstates Feature	Healthy:14, SZ:14	CNN	93
WeiKoh et al. [38] (2022)	Local configuration features of spectrograms of EEG	Healthy:14, SZ:14	KNN	97.2
Hassan et al. [39] (2023)	CNN based Feature Extraction	Healthy:14, SZ:14	Logistic Regression	98.05
T.Sunil Kumar et al. [12] (2023)	SLBP + HLV	Healthy:14, SZ:14	AdaBoost	99.15
Proposed approach	Microstates Feature + EMD Feature	Healthy:14, SZ:14	Logistic Regression	100
		Healthy:32, SZ:32		90.7

Subsequently, a comprehensive summary of the majority of recent studies on schizophrenia classification utilizing public Dataset-1 is presented, along with an overview of select studies based on other datasets (Table 4). Regarding EMD features, the comparative results with the literature [14] indicate that while previous studies employ complex entropy features derived from MEMD, this research achieves an accuracy of approximately 96% using traditional feature extraction methods. This finding suggests that high classification performance can be maintained without necessitating feature extraction steps characterized by high computational complexity. This result holds substantial significance, particularly in clinical applications, where the simplification of feature calculation facilitates the enhancement of feasibility and practicality for real-time detection in clinical practice. Regarding microstate features, our findings indicate an accuracy of 96%, which is comparable to the results reported in the literature [15], further substantiating the efficacy of microstate features in detecting schizophrenia. While the literature [15] does not offer additional evaluation metrics, the findings suggest that leveraging microstate features can yield classification performance on par with more complex methodologies. Moreover, these features have been rigorously validated for their clinical relevance [40,41,42], thereby enhancing both the interpretability and potential applicability of our detection method in clinical contexts. In conclusion, the fusion feature strategy introduced in this study demonstrates remarkable accuracy across numerous schizophrenia classification studies utilizing Dataset-1, while also maintaining robust performance metrics in Dataset-2. This effectively substantiates the efficacy of the proposed feature-level fusion approach.

### 3.2. Classifying the Severity of Schizophrenia Symptoms

In our investigation of the classification of schizophrenia symptom severity, we compared the evaluation metrics associated with various feature sets (EMD features, microstate features, and feature-level fusion strategies) across Logistic Regression classifiers. The SHAP algorithm is employed to elucidate the specific contributions of microstate features and EMD features in differentiating the severity of symptoms.

#### 3.2.1. Results and Analysis of the Evaluation Metrics

When using the micro-state features and EMD features separately, it was observed that the accuracy rates of the classifiers were 77.5% and 63.5%, respectively. Although theoretically, EMD features can capture the inherent nonlinearity and non-stationarity characteristics of complex electroencephalogram signals, the results (Table 5) indicate that their classification performance is significantly insufficient. The recorded AUC was only 0.799, and the ACC was only 0.635. Moreover, when using EMD features alone, the classification performance of the classifier was significantly lower than that of the micro-state features. This suggests that although EMD features can capture the nonlinear components of the electroencephalogram signals, their discriminatory ability remains relatively limited in different symptom categories of schizophrenia.

The microstate features exhibit significant classification advantages, with an AUC value close to 90% and an accuracy rate (ACC) exceeding 75%. All the indicators of the microstate features are superior to those of the EMD features in the classifier. The advantage of microstate features may stem from their direct reflection of the fundamental pathological mechanisms underlying schizophrenia, such as disruptions in brain functional networks and abnormalities in cognitive processing, which offer more explanatory discriminative information for classification models. Microstate features are more effective in capturing spatiotemporal patterns within EEG signals, which exhibit significant differences across varying levels of schizophrenia severity [43], thereby offering enhanced discrimination in classification tasks. Moreover, the computational complexity of microstate features is relatively low, facilitating their integration with traditional machine learning methods, which further enhances performance and operability in practical applications.

The microstate features and EMD features are subsequently fused into the classifier. In Feature-level Fusion, the proposed method achieves optimal accuracy in the Logistic Regression classifier (93.6%). The utilization of fused features significantly outperforms the use of any individual feature in the evaluation metrics, demonstrating that this fusion method effectively integrates the advantages of EMD features and microstate features, thereby enhancing the robustness and accuracy of the model. Simultaneously, this study posits that the feature fusion strategy not only preserves the spatiotemporal stability of microstate features but also integrates the nonlinear advantages of EMD features to construct a more comprehensive feature representation.

**Figure 3:**
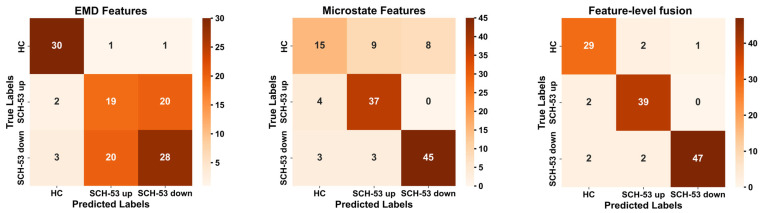
Confusion matrix of the logistic regression classifier for three features.

The confusion matrix incorporating EMD features, microstate features, Feature-level Fusion across classifiers is presented in Figure 3. We observed that EMD features excel in identifying healthy individuals but demonstrate reduced sensitivity in recognizing schizophrenia patients. This may suggest that EMD features more effectively capture the patterns of normal EEG while exhibiting certain limitations in detecting abnormal patterns. In contrast, microstate features perform exceptionally well in distinguishing the severity of symptoms among schizophrenia patients, indicating that these features can effectively capture characteristics and dynamic changes associated with the disorder. To further clarify the specific functions of these features, this study conducted SHAP analysis on the feature-level fusion features.

#### 3.2.2. SHAP-Based Interpretation of Feature Importance

In this section, we assessed the significance of features using SHAP values and examined the specific contributions of microstate features and EMD features to the classification of schizophrenia symptoms. Feature numbers f1 to f24 correspond to microstate features, while feature numbers f25 and beyond represent EMD features, as detailed in Appendix A Table A1.

**Figure 4:**
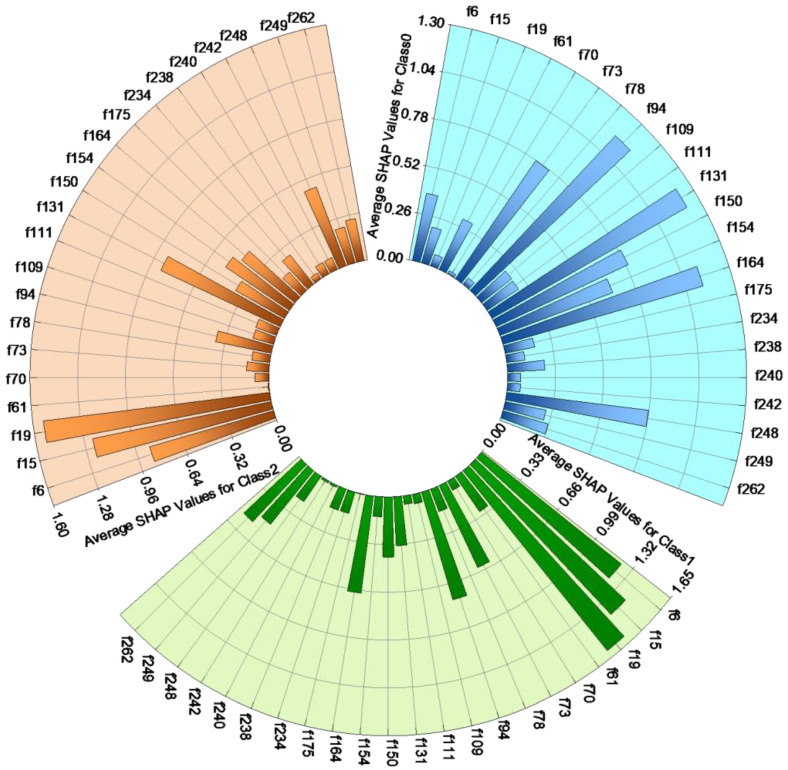
Impact of Various Features on the Output of the Logistic Regression Classifier. The entire image is presented in a clockwise orientation, illustrating the SHAP values of microstate and EMD features during the Logistic Regression classification process (the blue background represents Class 0: healthy individuals; the green background indicates Class 1: BPRS score > 53; and the orange background denotes Class 2: BPRS score < 53). Longer bars signify larger SHAP values for the respective features, while the color of the bars does not convey any discrimination. f1 to f24 represent microstate features, whereas f25 to fn (*n* > 25) denote EMD features. A comprehensive overview of the relationship between feature numbers and their corresponding names is presented in Appendix A Table A1.

In the Logistic Regression classifier under Feature-level fusion, SHAP value analysis (Figure 4) revealed that in Class 0 (healthy controls), three EMD features—f131 (1.221), f94 (1.156), and f164 (1.134)—demonstrated high importance. These findings indicate a potentially significant association between these features and EEG signal patterns in healthy controls. In Class 1 (patients with severe schizophrenia), microstate features f19 (1.594), f15 (1.423), and f6 (1.236) exhibited a strong influence, underscoring the pivotal role of these features in differentiating patients with severe schizophrenia. Similarly, in Class 2 (patients with mild schizophrenia), features such as f19 (1.528), f15 (1.221), and f6 (0.863) demonstrated significance. This indicates that these consistent microstate features play a pivotal role in differentiating individuals with schizophrenia across varying severity levels.

Upon analyzing the results of this fusion scheme, we observed the predominant role of microstate features in the classification task, particularly noting that features f19, f15, and others are critical for differentiating between varying severity levels of schizophrenia. This finding aligns with the existing literature [44] that underscores the dynamic changes in microstates observed in patients with schizophrenia. The higher SHAP values of these features indicate that microstate features are more effective than exclusively relying on EMD features in capturing the specific pathological characteristics of individuals with schizophrenia. As feature f19 represents the transition probability from microstate class B to microstate class D, it serves as a specific indicator of dysfunctional dynamic brain network connectivity in schizophrenia. It signifies a disruption in the fundamental “perception-action transition chain,” thereby providing a strong neurophysiological explanation for core negative symptoms and behavioral impairments in patients, such as delayed responses, lack of motivation, and social deficits. Meanwhile, feature f15 represents the transition probability from microstate class D to microstate class A, reflecting an abnormal dynamic pattern in the brain’s switch from a cognitive control state to an auditory and language processing state. This abnormal switching may be associated with thought disorders [36]. Furthermore, the EMD feature demonstrated greater significance in differentiating healthy controls, highlighting its enhanced relevance in analyzing normal EEG signals; however, its effectiveness in distinguishing the severity of schizophrenia was diminished.

Further analysis indicated that while microstate features demonstrated significant importance in differentiating among various categories of patients with schizophrenia, their relevance was comparatively lower in Class 0, which represents healthy controls. This implies that microstate features may be more adept at capturing the pathological characteristics associated with schizophrenia in studies examining the severity of the disorder. This implies that microstate features may be more adept at capturing the pathological characteristics associated with schizophrenia in studies examining the severity of the disorder. In contrast, EMD features demonstrated greater significance in Class 0, indicating that these features may be more adept at reflecting general neural activity patterns rather than specific pathological states. In EEG signal classification tasks, microstate features are more effective at capturing the neural dysfunctions associated with schizophrenia, while EMD features exhibit distinct advantages in identifying normal neural activity. The complementarity between these feature types provides substantial support for their synergistic effects in schizophrenia classification research.

In summary, the findings presented herein indicate that microstate features play a predominant role in detecting schizophrenia symptoms, while EMD features serve as a valuable complement in specific categories through feature combinations. The fusion of these two approaches offers theoretical and practical support for the multimodal classification of EEG signals and is anticipated to play an increasingly significant role in the classification of neuropsychiatric disorders in the future. Particularly in clinical applications, this multimodal feature fusion approach can enhance the model’s generalization ability across diverse patient populations, significantly improving both the robustness and accuracy of classification.

## 4. Conclusions

This study presents an innovative method for the intelligent diagnosis and assessment of symptom severity in schizophrenia, based on EEG signals, which fuses microstate analysis with EMD feature extraction techniques, significantly enhancing classification accuracy. The specific roles of feature-level fusion in assessing the severity of schizophrenia were also elucidated. Microstate features offer distinct advantages in capturing the spatiotemporal dynamic patterns of individuals with schizophrenia, whereas EMD features effectively characterize the nonlinear complexity inherent in EEG signals. The experimental results demonstrate that the fusion of these two approaches not only enhances the overall performance of the classifier, particularly in assessing symptom severity, but also achieves an accuracy of 90%, further validating the applicability and robustness of the proposed method.

Through a comprehensive evaluation of feature importance using SHAP analysis, we identified that microstate features and EMD features fulfill complementary roles across various classification tasks, thereby establishing a novel technical framework for accurately diagnosing schizophrenia. The findings of this study suggest that the feature extraction and fusion strategy of multi-channel EEG signals can enhance diagnostic accuracy and reliability, thus paving new avenues for early detection and personalized treatment of schizophrenia. Our future research will concentrate on further optimizing the feature selection and fusion algorithm, extending its application to larger and more diverse datasets, validating the universality and stability of this method across various clinical settings, and advancing the continuous development of diagnostic technology for schizophrenia.

## Figures and Tables

**Table 1:** Sample demographics of HC and SCH groups in Dataset-2.

	HC *n* = 32	SCH *n* = 92		F(T)/χ^2^	*p*
		SCH-53-up	SCH-53-down		
Sex:n (M:F)	17:15	21:20	24:27	0.326	0.849
Age, years: mean (SD)	24.91 (4.79)	27.44 (6.61)	25.12 (3.61)	0.533	0.588
BPRS: mean (SD)	-	55.61 (1.87)	47.22 (2.52)	17.58	0.000 ***

HC, healthy controls; SCH, schizophrenia patients; BPRS, Brief Psychiatric Rating Scale; SCH-53-up refers to schizophrenia patients with a BPRS score exceeding 53; SCH-53-down, schizophrenia patients with a BPRS score below 53; SD: Standard Deviation; The F-value is the statistical value of the one-way ANOVA test while the χ^2^-value is the statistical value of the chi-square. The T-values are statistics from paired sample *t*-tests. The significance criteria *p*-value is set at 0.05. Statistically significant differences are indicated in bold. *** *p* < 0.001.

**Table 2:** Results of schizophrenia detection using the Logistic Regression classifier on the Dataset-1. Here, all data represent the average of multiple experimental runs.

Features	AUC (95% CI)	SPE (95% CI)	SEN (95% CI)	ACC (95% CI)
EMD Features	1 (1.000–1.000)	0.950 (0.837–1)	1 (1.000–1.000)	0.966 (0.891–1)
Microstate Features	1 (1.000–1.000)	0.900 (0.674–1)	1 (1.000–1.000)	0.967 (0.891–1)
Feature-level fusion	1 (1.000–1.000)	1 (1.000–1.000)	1 (1.000–1.000)	1 (1.000–1.000)

**Table 3:** Results of schizophrenia detection using the Logistic Regression classifier on the Dataset-2. Here, all data represent the average of multiple experimental runs.

Features	AUC (95% CI)	SPE (95% CI)	SEN (95% CI)	ACC (95% CI)
EMD Features	0.917 (0.830–1)	0.917 (0.788–1)	0.875 (0.758–0.991)	0.893 (0.820–0.965)
Microstate Features	0.758 (0.588–0.928)	0.592 (0.343–0.841)	0.817 (0.701–0.931)	0.708 (0.580–0.834)
Feature-level fusion	0.972 (0.940–1)	0.908 (0.802–1)	0.908 (0.802–1)	0.907 (0.832–0.982)

**Table 5:** Evaluation index for severity classification of schizophrenia symptoms. Here, all data represent the average of multiple experimental runs.

Features	AUC (95% CI)	SPE (95% CI)	SEN (95% CI)	ACC (95% CI)
EMD Features	0.799 (0.714–0.884)	0.811 (0.737–0.885)	0.661 (0.524–0.799)	0.635 (0.496–0.775)
Microstate Features	0.873 (0.823–0.924)	0.886 (0.852–0.920)	0.752 (0.687–0.818)	0.775 (0.701–0.849)
Feature-level fusion	0.990 (0.978–1)	0.967 (0.950–1)	0.940 (0.905–1)	0.936 (0.900–1)

## Data Availability

The dataset we possess contains sensitive information, such as the fundamental demographic details of patients diagnosed with Schizophrenia. In accordance with the ethical agreement established with the hospital, please contact the corresponding author to request access to the dataset.

## Abbreviations

The following abbreviations are used in this manuscript:

EMD	Empirical Mode Decomposition
EEG	Electroencephalography
LASSO	Least Absolute Shrinkage and Selection Operator
SHAP	Shapley Additive Explanations
SCZ	Schizophrenia
BPRS	Brief Psychiatric Rating Scale
GFP	Global Field Power
IMF	Intrinsic Mode Functions

## Appendix A

**Table A1:** The relationship between feature numbers and their corresponding names.

Microstate Features		EMD Features	
Feature Number	Feature Name	Feature Number	Feature Name
f1	Mean duration class D	f25	Channel1-mean
f2	Mean duration class C	f26	Channel2-mean
f3	Mean duration class B	f27	Channel3-mean
f4	Mean duration class A	f28	Channel4-mean
f5	Time coverage class D	f29	Channel5-mean
f6	Time coverage class C	f30	Channel6-mean
f7	Time coverage class B	f31	Channel7-mean
f8	Time coverage class A	f32	Channel8-mean
f9	Occurrence class D	f33	Channel9-mean
f10	Occurrence class C	f34	Channel10-mean
f11	Occurrence class B	f35	Channel11-mean
f12	Occurrence class A	f36	Channel12-mean
f13	Transfer probability class D~C	f37	Channel13-mean
f14	Transfer probability class D~B	f38	Channel14-mean
f15	Transfer probability class D~A	f39	Channel15-mean
f16	Transfer probability class C~D	f40	Channel16-mean
f17	Transfer probability class C~B	f41	Channel17-mean
f18	Transfer probability class C~A	f42	Channel1-min
f19	Transfer probability class B~D	f43	Channel2-min
f20	Transfer probability class B~C	f44	Channel3-min
f21	Transfer probability class B~A	f45	Channel4-min
f22	Transfer probability class A~D	f…	…
f23	Transfer probability class A~C	f…	…
f24	Transfer probability class A~B	fn	Channeli-mk
